# A novel function of Prohibitin on melanosome transport in melanocytes

**DOI:** 10.7150/thno.41383

**Published:** 2020-03-04

**Authors:** Chan Song Jo, Hye In Park, Hyun Jin Jung, Jong il Park, Ji Eun Lee, Cheol Hwan Myung, Jae Sung Hwang

**Affiliations:** Department of Genetic Engineering & Graduate School of Biotechnology, College of Life Sciences, Kyung Hee University, Yongin, Gyeonggi-do 446-701, Republic of Korea.

**Keywords:** Prohibitin, melanosome transport, Rab27a, Melanophilin

## Abstract

Prohibitin (PHB, also known as PHB1 or BAP32), is a highly conserved 31kDa protein that expressed in many cellular compartments, such as mitochondria, nucleus, cytosol, and plasma membrane, and plays roles in regulating the transcription of genes, apoptosis, and mitochondrial biogenesis. There is a report that Prohibitin expression is required for the stimulation of pigmentation by melanogenin. However, no studies have been published on the function of PHB in melanocytes, especially in melanosome transport.

**Methods**: Immunofluorescence was performed to confirm the localization of PHB. *si*RNA transfections, Co-immunoprecipitation, western blotting and proximity ligation assay were performed to find binding state between proteins and demonstrate functions of PHB on melanosome transport.

**Results**: PHB is located in the melanosome and perinuclear aggregation of melanosome is induced when expression of PHB is reduced with no influence on melanin contents. PHB binds directly to Rab27a and Mlph but not Myosin-Va. Rab27a and Mlph bind to specific domains of PHB. Reduced expression of PHB led to the impaired binding affinity between Rab27a and Mlph.

**Conclusion**: PHB regulates melanosome transport by linking to Rab27a and Mlph in melanocytes. Targeting and regulating PHB not only manages pigmentation in melanocytes, but also controls hyperpigmentation in melanoma

## Introduction

Melanosomes, intracellular lysosome-related organelles, are a site for synthesis, storage, and transport of melanin pigments in skin melanocytes 1. In melanocytes, melanosomes are produced around the nucleus and mature through four stages of morphological change 2. Mature melanosomes can be moved along microtubules by dynein and kinesin motor proteins. When melanosomes reach the periphery of cells, they are released and move toward dendrites in a myosin-Va-dependent process, along with actin filaments, in a process called melanosome transport 3-6. Major proteins regulate actin-based melanosome transport, including Rab27a, Melanophilin and Myosin-Va. These proteins form a tripartite complex and play a role in regulating melanosome transport 6.

Rab27a is a member of the small GTPase Rab family that functions as a molecular switch between a guanosine triphosphate (GTP)-bound active form and a guanosine diphosphate (GDP)-bound inactive form 7-9. A guanine nucleotide exchange factor, Rab3GEF, is responsible for the activation of Rab27a in melanocytes. Rab27a regulates protein and melanosome transport 10,11. Melanophilin (Mlph, also known as Slac2a) is an effector protein of Rab GTPase protein that interacts with the GTP form of Rab27a and works as a linker between Rab27a and myosin-Va. Rab27a binds to SHD domains and Myosin-Va binds to MBD domains of Mlph 12-17. Myosin-Va is an ATP-dependent motor protein that transports melanosomes on actin filaments. The three proteins form a tripartite complex 17-21. If one of these proteins is decreased, perinuclear aggregation of melanosomes could occur because of the defect in melanosome transport from perinuclear sites to dendrite tips 21-23. Melanosome transport defects in mice, including homozygous mutation *dilute* (*Myosin-Va*), *ashen* (*Rab27a*) and *leaden* (*Mlph*), cause melanosomes to aggregate in the perinuclear region 24-26.

To find novel proteins which binds to Mlph, we set up an affinity proteomics system and found Prohibitin is one of the proteins that interacts with Mlph in melanocytes (data not shown). Prohibitin (PHB, formerly known as BAP32) and its homolog PHB2 (formerly BAP37, REA, or Prohibitone) are pleiotropic proteins with multiple functions. Over the last few years, PHBs have been found to interact with more than 60 other proteins to regulate a myriad of cellular events 27. PHBs have since been shown to act as a hub for many signaling pathways triggered by growth factors, the immune response, and steroid hormones regulating metabolism, mitochondrial biogenesis, cell migration, division, and survival. Both PHB and PHB2 are composed of an N-terminal transmembrane domain, an evolutionarily conserved PHB domain that is similar to that of lipid raft-associated proteins, and a C-terminal coiled-coil domain that is involved in protein-protein interactions, including the interaction between PHB and PHB2 as well as transcriptional regulation. PHB and PHB2 are interdependent on the protein level, and loss of one simultaneously leads to the loss of the other 28,29.

PHB is a shuttle protein that shuttles between subcellular compartments. Newly synthesized PHB and PHB2 in the cytoplasm or mitochondrially located PHB and PHB2 need to pass through the nuclear pore complex to translocate into the nucleus, where they act as a transcriptional modulator. It has been demonstrated that human PHB2 contains both an uncleavable mitochondrial targeting sequence (MTS) at its N terminus and a nuclear localization sequence at its C terminus 29. Although human PHB does not possess the MTS, the N-terminal portion of PHB is also the sole determinant of mitochondrial targeting 29. Moreover, the coiled-coil structure of the alpha helices in the C terminus contains a leucine/isoleucine-rich nuclear export sequence (NES) that facilitates the export of PHB to the mitochondria or cytoplasm 30. This specific structure of PHB proteins allows active shuttling between the organelles. Snyder *et al*. 31 identified melanogenin as an inducer of pigmentation. This compound upregulates tyrosinase, a rate-limiting enzyme in the biosynthesis of melanin. They conjugated melanogenin to an agarose support, which enabled them to identify PHB as the molecular target by affinity chromatography. Further biological investigations confirmed that PHB is responsible for the induction of pigmentation. They suggest that the binding of melanogenin to PHB may disrupt the interaction between PHB and a transcriptional factor, thereby causing its translocation to the nucleus and induction of the expression of tyrosinase, the rate-limiting enzyme in melanogenesis. Recently, Wu and Wu 32 demonstrated that PHB is translocated to lipid rafts in HaCaT keratinocytes after UVB irradiation, and that protects the cells against UVB-induced apoptosis. From these findings we can infer that PHB contributes to protection of the skin against UVB. However, there are no studies in the literature on the function of PHB on melanosome transport in melanocytes. 28, 33-38. In this study, we attempt to determine the function of PHB on melanosome transport.

## Results

### Localization of PHB in melanocytes

**Figure 1:**
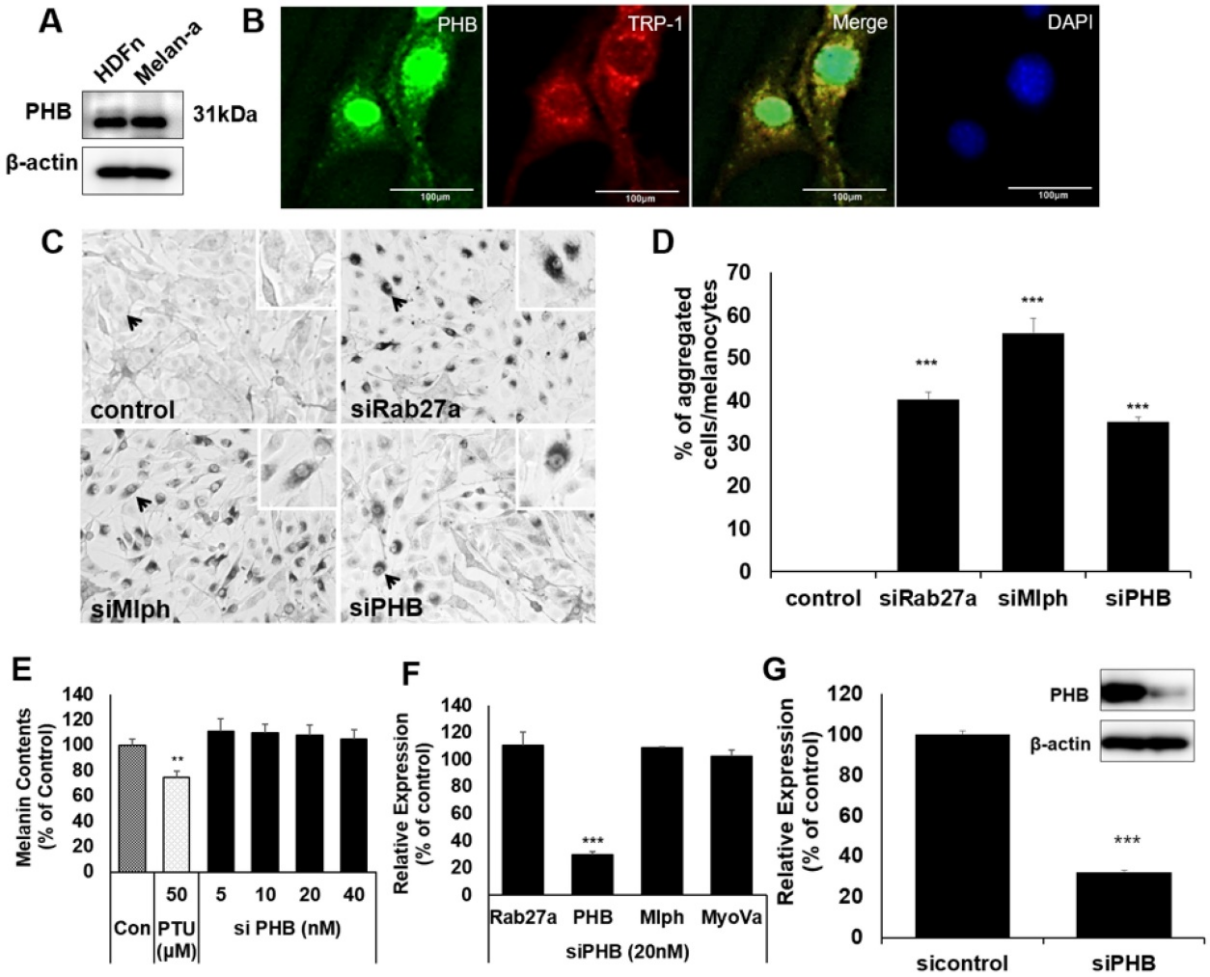
** A** IB analysis for PHB in Melan-a cells compared with normal human dermal fibroblast (HDFn). **B** Melan-a cells were stained with DAPI (blue) for nuclei, FITC (green) for PHB and PE (red) for TRP-1. PHB co-localized with TRP-1 (yellow) is shown. **C** Representative cell images transfected with Rab27a, Mlph, PHB of **si**RNAs. **D** Quantification of melanosome transport observed in the transfected cells. **E** Melanin contents derived from PHB-KD Melan-a cells. As indicated, cells were treated with PHB of *si*RNAs (20 nM) for 72h. **p < 0.01. **F** mRNA levels derived from PHB-KD Melan-a cells. ***p < 0.001. **G** Protein levels derived from PHB-KD Melan-a cells. ***p<0.001.

There are few studies on PHB with melanin, and melanocytes. We confirmed that PHB is expressed in Melan-a mouse melanocytes (Figure 1A).

To confirm whether PHB is localized in melanosomes, we conducted immunofluorescence staining with PHB and TRP-1 antibodies as melanosome markers. Fluorescence microscopy revealed that PHB was co-localized with TRP-1 (Figure 1B). When PHB expression was reduced by transfection of *si*PHB, perinuclear aggregations of melanosome were observed, such as a defect in *Rab27a* or *Mlph* (Figure 1C-D). To assess the impact of PHB on melanin synthesis, we transfected *si*PHB into Melan-a cells. Three days after transfection of *si*PHB, melanin contents were unchanged (Figure 1E). The mRNA level of Rab27a, PHB, Mlph, and Myosin-Va was measured by quantitative polymerase chain reaction (qPCR) analysis after *si*PHB (20nM) treatment in Melan-a cells (Figure 1F). When mRNA levels for PHB were reduced by transfection of siPHB, the mRNA level of Rab27a, Mlph and Myosin-Va was not affected. The protein level of PHB was measured by western blot after *si*PHB (20nM) treatment in Melan-a cells (Figure 1G).

### PHB binds directly to Rab27a and Mlph, not Myosin-Va

**Figure 2:**
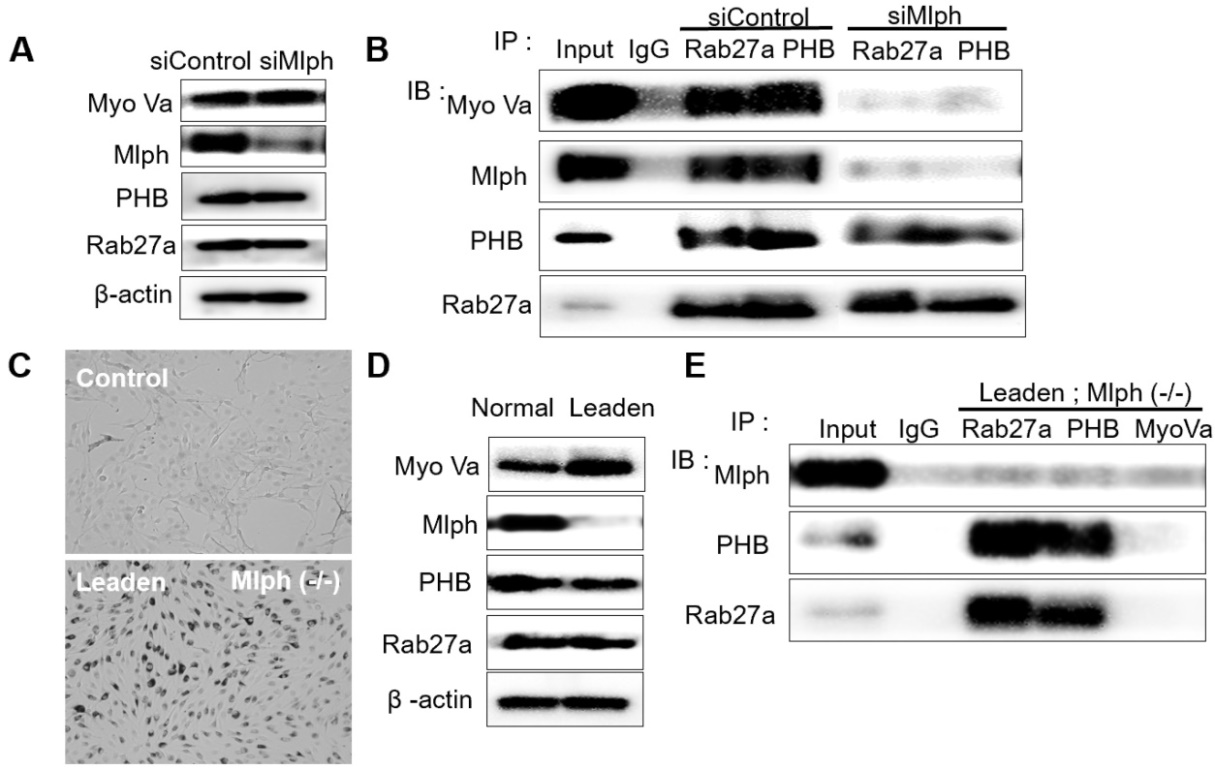
** PHB is bound directly to Rab27a in Melan-a cells. A** IB analysis for Rab27a, PHB, Mlph, and Myosin-Va of Mlph-KD Melan-a cells were treated with Mlph of siRNAs (20 nM) for 72h. **B** Anti-Rab27a and PHB immunoprecipitated (IP) cells. As indicated, were performed for O/N at 4 °C followed by western blot analysis to check co-immunoprecipitated (co-IP) proteins (WB). Whole cell lysates were analyzed for total protein levels. **C** Representative cell images compared *Leaden* Melan-a cells with control, Melan-a cells. **D** IB analysis for Rab27a, PHB, Mlph, Myosin-Va of *Leaden* Melan-a cells. **E** Anti-Rab27a, PHB, and Myosin-Va IP were performed for O/N at 4 °C followed by western blot analysis to check co-IP proteins (WB). Whole cell lysates were analyzed for total protein levels.

To investigate whether PHB interacts with Rab27a, co-immunoprecipitation was performed with whole Melan-a cell lysates in which Mlph expression was reduced by *si*Mlph. Melan-a melanocytes were cultured and transfected for 72 h with 20 nM of *si*Mlph. When Mlph expression was decreased by transfection of *si*Mlph, the expression of Rab27a, PHB, and Myosin-Va was not affected (Figure 2A). By co-immunoprecipitation (co-IP), we found that PHB binds to Rab27a (Figure 2B). To confirm the binding of PHB and Rab27a, we conducted the same experiment using *Leaden* Melan-a cells, which are Mlph mutants. Melanosome aggregation at the perinuclear site occurred in *Leaden* cells (Figure 2C). Mlph expression was reduced in *Leaden* cells, without changing the expression of Rab27a, PHB, or Myosin-Va (Figure 2D). By co-immunoprecipitation, we also found that PHB bound to Rab27a in the absence of Mlph (Figure 2E). Taken together, we found that PHB interacts with Rab27a.

**Figure 3:**
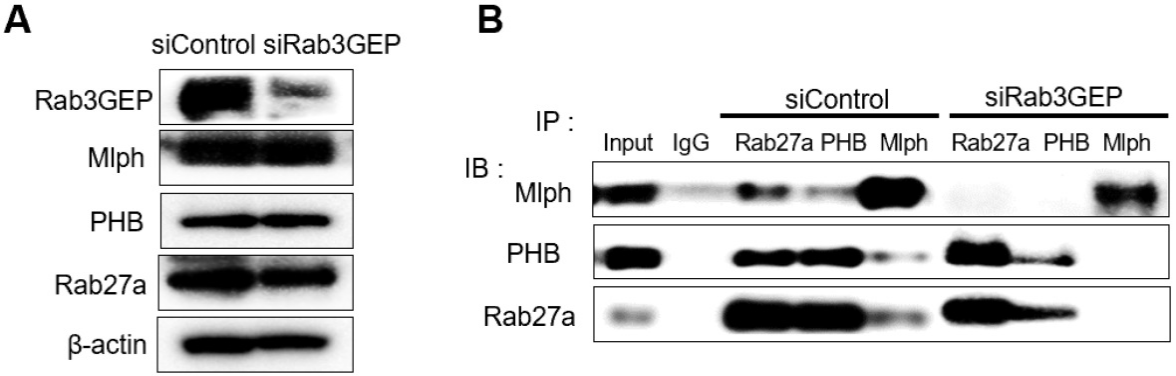
** PHB is bound to GTP and GDP-bound form of Rab27a. A** IB analysis for Rab27a, PHB, Mlph, and Rab3GEP (MADD) of Rab3GEP-KD Melan-a cells. As indicated, cells were treated with Rab3GEP of siRNAs (50nM) for 72h. **B** Anti-Rab27a, PHB, and Mlph immunoprecipitated (IP) were performed for O/N at 4 °C followed by western blot analysis to check co-immunoprecipitated proteins (WB). Whole cell lysates were analyzed for total protein levels.

Next, we tried to determine whether the binding of PHB to Rab27a depends on a nucleotide switch form of Rab27a. Melan-a cells were transfected with 50 nM *si*RNA specific for Rab3GEP to make inactive GDP-bound form of Rab27a. Then, we observed that Rab3GEP expression was decreased and did not affect other proteins, such as Rab27a, Mlph, and PHB (Figure 3A). As a consequence of co-immunoprecipitation with whole cell lysates of Rab3GEP-KD, we discovered that PHB bound to the inactive GDP-bound form of Rab27a as well as the active GTP-bound form (Figure 3B). From these results, we found that PHB could interact with active and inactive form of Rab27a, unlike Mlph which interacted with the active form of Rab27a only. But more direct evidence should be needed to confirm these results.

**Figure 4:**
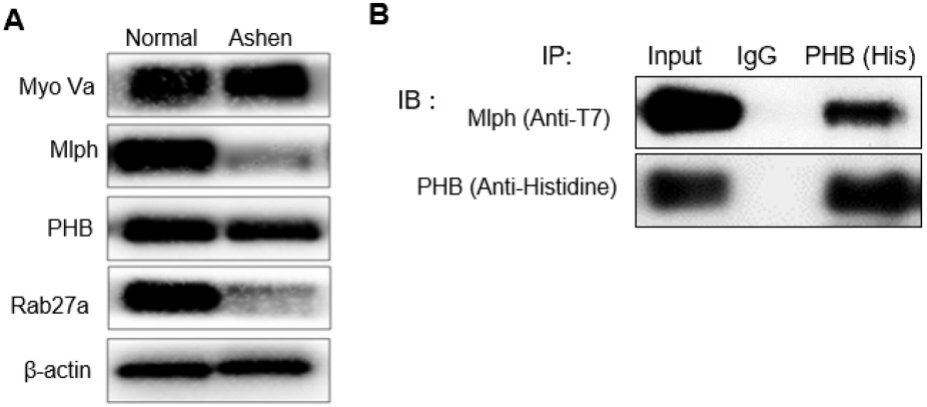
** PHB is bound directly to Mlph in Melan-a cells. A** IB analysis for Rab27a, PHB, Mlph, and myosin-Va of *Ashen* Melan-a cells. **B** COS7 monkey kidney cells were transfected with PHB and Mlph plasmids. Anti-histidine immunoprecipitated (IP) were performed for 3 h at 4 °C followed by western blot analysis to check co-IP (WB). Whole cell lysates were analyzed for total protein levels.

We then investigated the interaction between PHB and Mlph. The knockdown of Rab27a by treatment with *si*Rab27a induced proteasomal degradation of Melanophilin 39. In *ashen*, *Rab27a* mutant Melan-a melanocyte cells, we confirmed that Mlph expression was also reduced (Figure 4A).

Using COS7 monkey kidney cells that do not express Rab27a and Mlph, we constructed expression plasmids of PHB and Mlph, and transfected them into the COS7 cells (Figure S1A). We then conducted co-immunoprecipitation with the transfected COS7 cells and found that PHB attached to Mlph (Figure 4C).

From these data, binding between proteins could be established, but we did not confirm direct binding between proteins. We therefore performed a proximity ligation assay (PLA) to verify direct binding of PHB and Rab27a. The PLA depends on the dual proximal binding by pairs of detection reagents to generate amplifiable DNA strands, which then serve as surrogate markers for the detected protein molecules. The oligonucleotides on the proximity probes, when brought into close proximity by binding adjacent proteins, serve as templates for the circularization of so-called connector oligonucleotides by enzymatic ligation. PLA confers dual-binder specificity for protein detection in situ and can reveal interactions between proteins directly in normal cells and tissues without being subject to artifacts of overexpression or ectopic expression 40.

**Figure 5:**
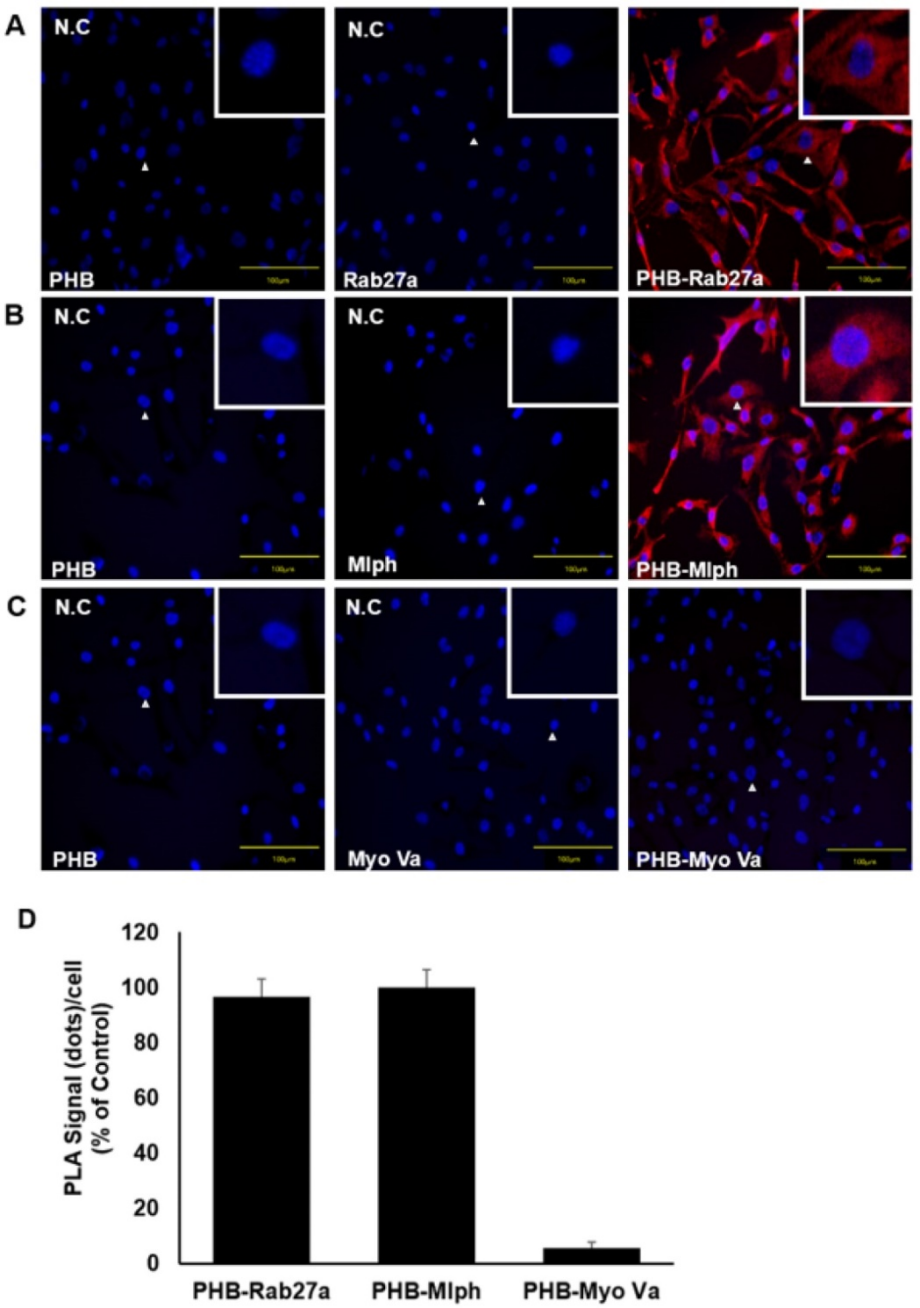
** PHB is bound directly to Rab27a and Mlph, not myosin-Va. A, B, C** PLA assay was performed with Melan-a cells stained with DAPI (blue) for nuclei and PLA signals (red) indicated protein interaction events (scale bar=100 µm). **A** Representative images of the PHB-Rab27a complex. For a negative control, the cells were stained with anti-Rab27a or PHB IgG antibodies, only. **B** Representative images of PHB-Mlph complex. For negative controls, the cells were stained with anti-Mlph or PHB-immunoglobin G (IgG) antibodies, only. **C** Representative images indicating an absence of PHB-Myosin Va interaction. For negative controls, the cells were stained with anti-Myosin-Va or PHB-IgG antibodies, only. **D** Quantification of PLA data (mean ± standard deviation, *n* = 3).

We observed a direct interaction between PHB and Rab27a in Melan-a cells (Figure 5A) and found that PHB bound to Rab27a, independent of Mlph. A PLA conducted to verify direct interaction between PHB and Mlph in Melan-a cells (Figure 5B) found that PHB also bound to Mlph, independent of Rab27a. To confirm the interaction between PHB and Myosin Va, we performed a proximity ligation assay in Melan-a melanocytes but did not observe a direct interaction between PHB and Myosin Va (Figure 5C). These data provide compelling evidence that PHB binds directly to Rab27a and Mlph, but not to Myosin Va (Figure 5D).

### Rab27a and Mlph bind to a specific domain of PHB

**Figure 6:**
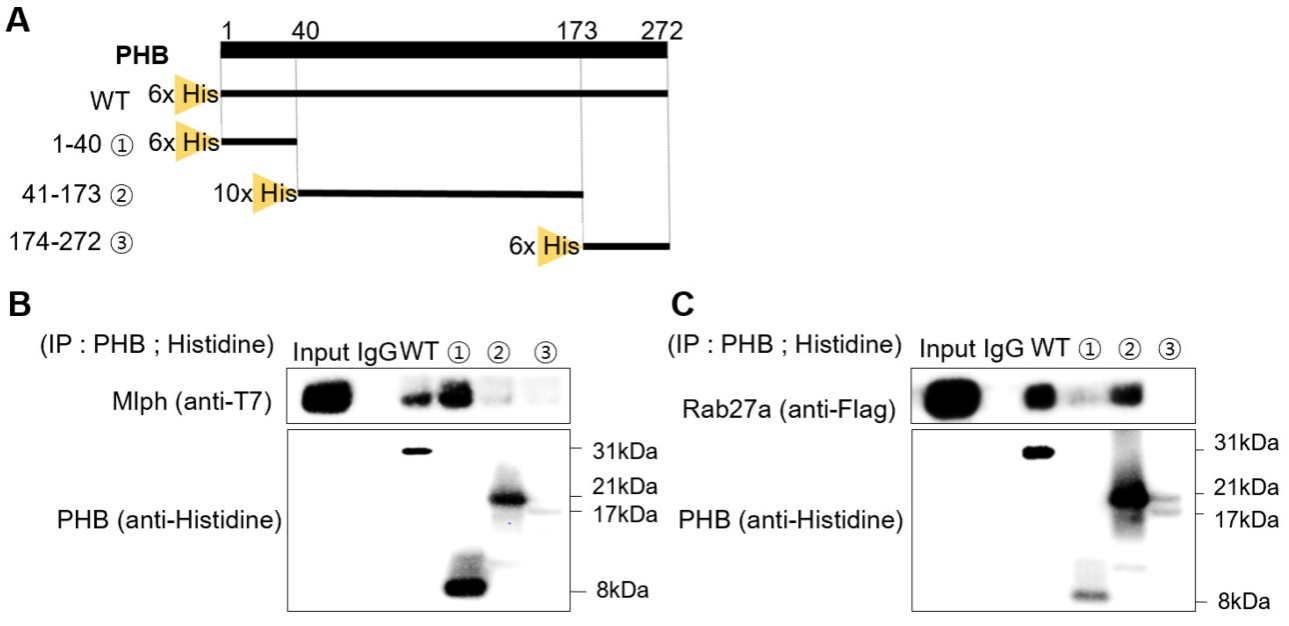
** Rab27a and Mlph are bound to specific domains of PHB. A** Alignment of PHB sequences (mouse) that PHB domains present for a transmembrane domain, a prohibitin domain, and a coiled-coil domain. **B, C** Anti-histidine pull-down immunoprecipitation was performed with Mlph-transfected-**(B)** and Rab27a transfected-**(C)** cells for 1 h at 4 °C followed by western blot analysis to check immunoprecipitated proteins (WB). Rab27a transfected Melan-a cells were treated with proteasome inhibitor MG132 (1 µM) for 3 h before cell collection.

To find the domain of PHB where Rab27a and Mlph are bound, we cut mouse PHB into three domains and expressed the domains separately. The first domain is the transmembrane domain, with amino acids 1-40. The second domain is the prohibitin domain, with amino acids 41-173. The third is the coiled-coil domain, with amino acids 174-272 (Figure 6A). We constructed expression plasmids of Mlph and Rab27a, and then transfected the plasmids into the COS7 cells (Figure S1B-C). With these three domains, we conducted a pull-down assay to determine which PHB domain could bind to Mlph. PHB (wild type or domain) was pulled down with Mlph-transfected COS7 cell lysates using a histidine antibody. Mlph was bound to the transmembrane domain of PHB with amino acids 1-40 (Figure 6C). Next, we conducted a pull-down assay to determine which domain of PHB could bind to Rab27a. PHB (wild type or domain) was pulled down with Rab27a-transfected COS7 cell lysates again using a histidine antibody. We determined that Rab27a was bound to the prohibitin domain of PHB with amino acids 41-173.

### Function of PHB on melanosome transport: PHB is required for the interaction between Mlph and Rab27a

**Figure 7:**
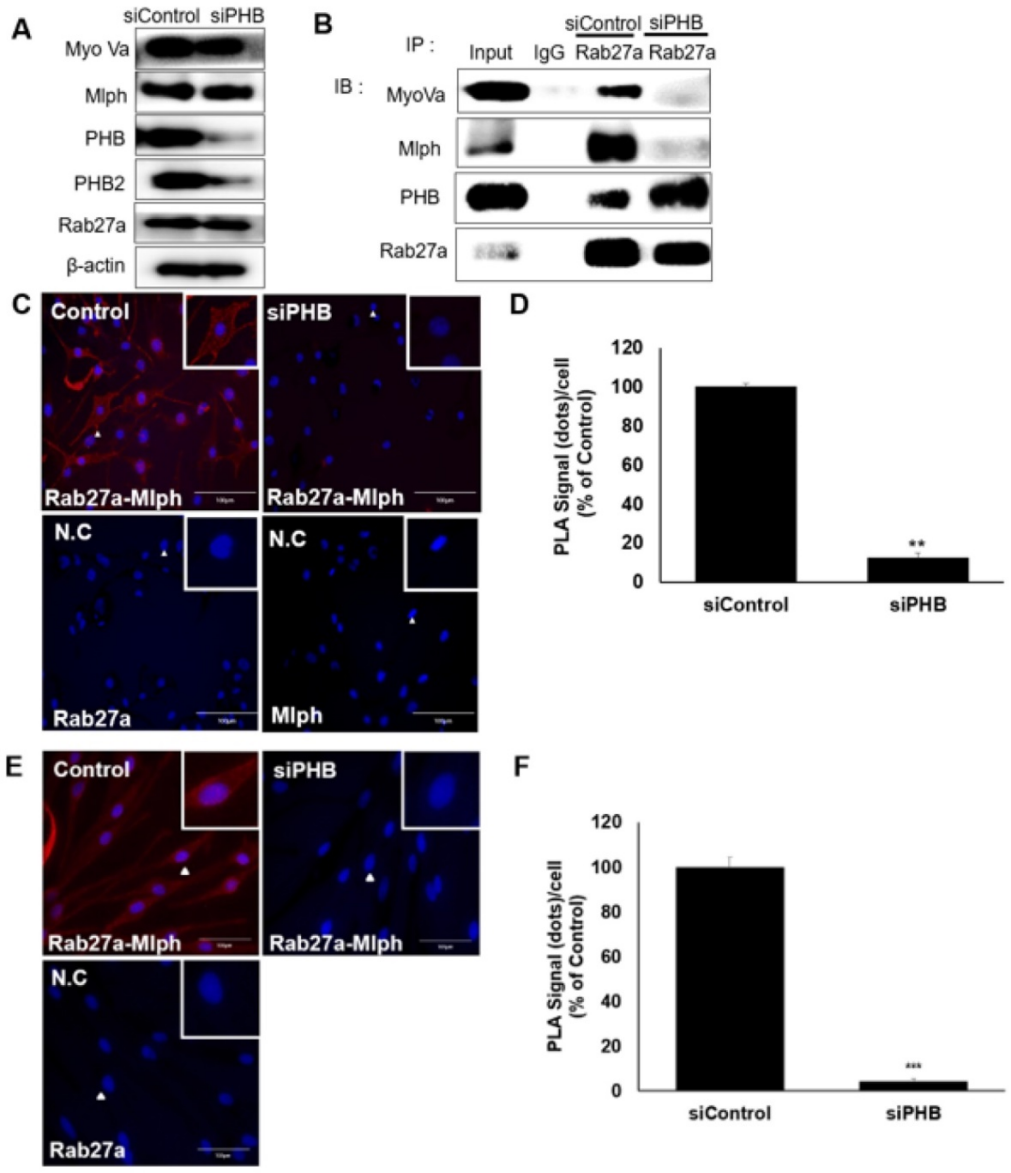
** PHB is necessary for binding of Rab27a and Mlph. A** IB analysis for Rab27a, PHB, Mlph, and myosin-Va after transfection of *si*PHB (20 nM) for 72h. **B** Anti-Rab27a immunoprecipitated (IP) was performed for O/N at 4 °C followed by western blot analysis to check co-IP proteins (WB). Whole cell lysates were analyzed for total protein levels. **C, D** PLA assay was performed with control and PHB-KD Melan-a cells (scale bar=100 µm). PLA signals (red) indicated Rab27a-Mlph interaction events. The cells were stained with DAPI to supply evidence for the nuclei. For negative control, the cells were stained with anti-Rab27a or Mlph-IgG antibodies only (**C** representative picture, **D** quantification)/ (mean ± standard deviation, *n* = 3). **p < 0.01. **E,F** PLA assay was performed with control and PHB-KD NHEM cells (scale bar=100 µm). PLA signals (red) indicated Rab27a-Mlph interaction events. The cells were stained with DAPI to supply evidence for the nuclei. For negative control, the cells were stained with anti-Rab27a or Mlph-IgG antibodies only (**E** representative picture, **F** quantification) (mean ± standard deviation, *n* = 3). ***p < 0.001.

We investigated the role of PHB in the tripartite complex. When Melan-a melanocytes were transfected with *si*RNA specific to PHB, the expression of PHB decreased without affecting other proteins, such as Rab27a, Mlph, or Myosin Va (Figure 7A, S2) But the interaction of Rab27a and Mlph was decreased and detected by a co-immunoprecipitation assay with whole cell lysate of PHB-KD (Figure 7B). To confirm of the role of PHB, we performed a proximity ligation assay. No interaction between Rab27a and Mlph was detected in PHB-KD cells compared with controls in Melan-a cells (Figure 7C-D). Loss of interaction between Rab27a and Mlph was also detected in PHB-KD normal human epidermal melanocytes (NHEM) (Figure 7E-F). Taken together, these data indicate that Mlph and Rab27a, which are important to melanosome transport, are connected by PHB.

## Discussion

We employed an affinity purification method to identify interacting partners of Mlph, which is a linker protein in the melanosome transport complex. One of the identified proteins was Prohibitin, but its function in melanosome transport has not been reported. Using proteomic analysis of murine skin, Huang *et al* (2003) showed that an increased level of Prohibitin proteins were present in epidermal cells compared to subepidermal tissues 41.

In dendritic cells, Rab32/38, a member of the mammalian Rab GTPase family, forms a complex with two interacting proteins, PHB and PHB2, and regulates intracellular bacterial proliferation 42. Rab32/38 also regulates the biogenesis of melanosomes in melanocytes and is required for trafficking of Tyrosinase and Tyrp1 from early to later stages of melanosomes in melanocytes 43. Unlike Rab32/38, Rab27a is involved in the later stages of melanosomes in melanocytes and regulates melanosome transport.

PHB works with many target proteins in various cellular compartments. PHB interacts with Raf and is required for Ras-induced Raf/MAPK/ERK activation by the epidermal growth factor receptor and modulates epithelial cell adhesion and migration 44, 45. PHB is also found in the mitochondrial inner membrane and forms a complex with VDAC, ANT2 and HAX-1, which are involved in apoptosis 46. PHB is a transcriptional corepressor for E2F1 and arrests proliferation 47.

Recently, it has been reported that Prohibitin/Annexin2 interaction regulates fatty acid transport in adipose tissue 48. Fatty acid uptake stimulates assembly of a complex of PHB, ANX2, and CD36 and the complex regulates endothelial fatty acid transport into adipocytes. These results show that PHB may be involved in complex formation and has a role in regulating transport.

We showed that PHB is expressed in Melan-a mouse melanocytes (Figure 1A) and is co-localized with TRP-1, a melanosome marker (Figure 1B). Perinuclear aggregation of melanosomes was detected in PHB-KD Melan-a cells like Rab27a-KD and Mlph-KD (Figure 1C), but transfection of *si*PHB had no influence on melanin contents (Figure 1D-E). Based on these findings, we hypothesized that PHB may regulate melanosome transport by binding to the tripartite complex. We found that PHB interacts with Rab27a (Figure 2, 5A) and Mlph (Figure. 4, 5B) independently. The interaction of PHB and Rab27a was not influenced by the GDP or GTP form of Rab27a (Figure 3). There was no interaction with PHB and Myosin-Va (Figure 5C). We found that Mlph was attached to the transmembrane domain of PHB, and Rab27a was attached to the prohibitin domain (Figure 6). Finally, interaction between Mph and Rab27a was decreased by treatment of *si*PHB in Melan-a cells (Figure 7). From these results, we concluded that PHB strengthens or sustains the interaction between Mlph and Rab27a.

We demonstrated a role for PHB in melanosome transport that is necessary for interactions between Rab27a and Mlph of the Rab27a/Mlph/Myosin-Va tripartite complex. Specific and druggable targets for the treatment of skin hyperpigmentation are important, such as CREB-regulated co-activator 1^49^. The novel role of PHB in melanosome transport may prove useful in the study of skin pigmentation, may supply a novel method of controlling skin pigmentation, and may be the basis for the development of pigment modulators. Targeting and regulating PHB not only manages pigmentation in melanocytes, but also controls hyperpigmentation in melanoma. Rab27a has been described as a driver gene in melanoma 50,51 and recently, Guo D *et al.* 52 reported that Rab27a was overexpressed in a subset of melanomas and Rab27a promotes melanoma cell invasion and metastasis via the secretion of pro-invasive exosomes. Even more, Guo D *et al.* 53 found the interruption of Rab27a and Mlph binding reduced melanoma cell motility and invasion. So, understanding the role of PHB on the binding of Rab27a and Mlph can give a new insight on melanoma progression and targets for treatment. Defining the biochemistry of Rab27a/PHB/Mlph/Myosin-Va interactions on the melanosomes mediating their transport will lead to a better understanding of the mechanisms governing melanocytes and skin pigmentation.

## Methods

### Cell culture

Melan-a melanocytes are normal, immortalized murine melanocyte cells derived from C57BL/6 mice. Melan-a melanocytes were obtained from Dorothy Bennett (St. George's Hospital, London, UK). Melan-a melanocytes were maintained in a RPMI-1640 (Welgene, Korea) medium supplemented with 10% fetal bovine serum (FBS), 100 U/mL penicillin, 100 µg/mL streptomycin, and 200 nM phorbol 12-myristate 13-acetate (PMA; Sigma-Aldrich, St. Louis, MO). Melan-a *Leaden* (or* Ashen*) melanocytes are immortalized *Melanophilin* (or *Rab27a*) mutant murine melanocyte cells derived from C57BL/6J 54. *Leaden* and *Ashen* melanocytes were obtained from Dorothy Bennett (St. George's Hospital, London, UK). Cells were maintained in RPMI-1640 (Welgene, Korea) medium supplemented with 10% fetal bovine serum (FBS), 100 U/mL penicillin, 100 µg/mL streptomycin and 200 nM PMA and 2 nM cholera toxin (Sigma-Aldrich, St. Louis, MO). HDFn cells were maintained in Dulbecco's Modified Eagle Medium (Welgene, Korea) supplemented with 10% FBS, 100 U/mL penicillin, and 100 µg/mL streptomycin. COS7 monkey kidney cells were maintained in SMEM (Sigma-Aldrich, St. Louis, MO) supplemented with 10% FBS, 100 U/mL penicillin, and 100 µg/mL streptomycin. NHEM cells were maintained in a Medium 254 (gibco) supplemented with 1%HMGS, 100 U/mL penicillin, and 100 µg/mL streptomycin.

### Small interfering RNAs (*si*RNAs)

*si*RNA oligonucleotides including negative control were purchased from Bioneer. Sense and antisense sequences for individual duplexes targeting mouse Rab27a, Mlph, PHB, Rab3GEP were as follows: Rab27a sense 5'-GGAGAGGUUUCGUAGCUUAUUTT-3'; anti-sense 5'-UAAGCUACGAAACCUCUCCUUTT-3'; Mlph sense 5'-ATGTAGACACCTCTGATGAAGA-3'; anti-sense 5'-TTAGGGCTGCTGGGCCATCAC-3'; Prohibitin sense 5'- GCAUUGGCGAGGACUAUGAUU-3'; anti-sense 5'-UCAUAGUCCUCGCCAAUGCUU-3'; another Prohibitin (Supplementary data 2) sense 5'-CGTGGAAGGCGGTCATAGAGC-3'; anti-sense 5'-TCGGGACAGCACACGCAGGGAGAT-3'; Rab3GEP sense 5'-ACTCCCAGACCTTATTTCCA-3'; anti-sense 5'-CAAGATGATCAGCACCTTAGC-3'.

### *si*RNA transfection

One day before transfection, Melan-a melanocytes were seeded in six-well plates at 60% confluency with 20 nM *si*RNAs mixed with Lipofectamine RNAiMAX Transfection Reagent (Invitrogen, CA) and treated according to the manufacturer's protocol. After three days, protein was obtained. Rab3GEP *si*RNAs were treated in 50 nM.

### qPCR

Quantitative real-time PCR was performed using a FastStart Essential DNA Probes Master kit (Roche, Mannheim, Germany) with Universal ProbeLibrary (Roche). The reaction was carried out according to the manufacturer's protocol. The probes for *Rab27a* (#63, NM_023635.6), *Mlph* (#108, NM_053015.3), *Myo Va* (#63, NM_010864.2) and *Phb* (#34, NM_008831.4) were designed by the Probe Library Assay Design Center. The cycling condition was annealing for 600s at 95 °C and 40 cycles at 95 °C for 20 s and 60 °C for 40 s on Lightcycler® Nano. Expression level of two genes was normalized to mouse β-actin, a control gene. The obtained cDNA was amplified with the following primers: *Rab27a* sense 5'-GAAGACCAGAGGGCAGTGAA 3'; antisense 5'-ACTGGTTTCAAAATAGGGGATTC-3'; *Mlph* sense 5'-AGCCCCTCAACAGCAAAAA-3'; antisense 5'-TTCCTCAAAGTCCACATCTCG-3'; *Myo Va* sense 5'-GCGCCATCACCCTAAACA-3'; antisense 5'-CCAGTTGACTGACATTGTACCTG-3'; *Prohibitin* sense 5'-CTGACCTTCGGGAAGGAGTT-3'; antisense 5'- GGCTGCCTTCTTCTGCTG-3'.

### Melanin assay

Melan-a melanocytes were seeded in 24-well plates in RPMI 1640 medium containing 10% FBS, 1% P/S and 200 nM PMA for 72h. After washing with Dulbecco's phosphate-buffered saline (PBS), the cells were dissolved in 100 µL of 1N NaOH at 55 °C for 30 min. Then, the lysates were measured at 450 nm using a spectrophotometer. The data were normalized to the protein contents of the cell lysates. The cell lysates were subsequently processed for the determination of the protein concentration using a BCA Protein Assay Kit.

### Immunofluorescence

Melan-a melanocytes were fixed with 4% formaldehyde in PBS for 15 min, washed, and permeabilized with 0.5% Triton X-100 for 30min. The fixed cells were incubated with 1% bovine serum albumin in tris-buffered saline-Tween 20 (TBS-T) for 30 min and then were incubated overnight with gentle rocking at 4 °C with PHB or Rab27a antibodies. The cells were washed three times with PBS and then incubated for 30 min with fluorescein isothiocyanate to detect Rab27a or with phycoerythrin to detect PHB. After washing with PBS, fluorescence images were obtained with a fluorescence microscope.

### Proximity Ligation Assay

Duolink in situ proximity ligation analysis (PLA) 55 was performed following the manufacturer's instructions (Sigma, St. Louis, MO). Briefly, Melan-a melanocytes were grown on glass coverslips, fixed with 4% formaldehyde in Dulbecco's PBS and 0.1% Triton X-100 in PBS, and then blocked using Duolink blocking solution. The primary antibodies (1:50) were incubated at 4 °C, overnight. Secondary antibodies (Duolink In Situ PLA probe anti-rabbit plus and Duolink In Situ PLA probe anti-mouse minus) were then added, followed by the addition of the ligation and amplification solution. Mounting 4′,6-diamidino-2-phenylindole medium was treated and PLA signals were detected with fluorescence microscopy. The number of PLA signals per cell was quantified by counting the number of red spots in 20 cells with image software 'ImageJ'.

### Detection and quantification of melanosome aggregation

Melan-a melanocytes were seeded in 24-well plates and maintained for 24 h. The cells were then rinsed in DPBS and treated with samples in RPMI-1640 containing 2% FBS for 3 days. The cells were observed in bright field using an Olympus CKX41 culture microscope (Olympus, Japan), and images were photographed using a DMC camera (INS Industry, Korea) and DMC advanced software adapted to the microscope. Evaluation of melanosome aggregation was performed by counting cells with perinuclear melanosome aggregates in three random microscopic fields per well at x200 magnification. Values are presented as the mean ± SD from three wells (n = 3).

### Plasmid construction

pET28a-6xHis-PHB, pcDNA3.1-FLAG-Rab27a, and T7-Mlph were constructed. Mouse PHB and Rab27a were amplified by PCR and digested with BamH1 and EcoR1, then inserted into pET28a vector or pcDNA3.1 (+) containing 6x histidine or a FLAG tag. Construction of pET28a-6xHis-PHB was cloned by infusion with a vector after PCR using primers (forward; 5'-CGCGCGGCAGCCATATGGCTGCCAAAGTGTTTG-3', reverse; 5'-GGTGGTGGTGCTCGAGCTGGGAAGCTGGAGAAG-3'). Construction of pcDNA3.1-FLAG-Rab27a was cloned by infusion with a vector after PCR using primers (forward; 5'-TACCGAGCTCGGATCCGACTACAAAGACGATGAC-3', reverse; 5'-GATATCTGCAGAATTCTCAACAGCCACACAACCC-3'). Mouse Mlph was amplified by PCR and digested with EcoR1 and Xba1, then inserted into pcDNA3.1 (+) containing a T7 tag. Construction of pcDNA3.1-T7-Mlph was cloned by infusion with vector after PCR using primers (forward; 5'-GACGGCCAGTGAATTCATGGCATCAATGACAG-3', reverse; 5'-GGATCCGATATCTAGACTCGAGTTAGGGCTGC-3').

### Plasmid transfection

One day before transfection, COS7 monkey kidney cells were seeded in 100 mm dishes at 70% confluency and plasmids were mixed with Lipofectamine 2000 Transfection Reagent (Invitrogen, CA) and treated according to the manufacturer's protocol. In case of FLAG-Rab27a, 10µM of MG132 (Sigma-Aldrich, St. Louis, MO) was treated after 3 h of transfection and protein was obtained after 24 h.

### Co-immunoprecipitation

Equal amounts of whole cell extracts were rotated and immobilized in protein A plus agarose beads (Santacruz, Dallas, TX) with specific antibodies (1-2 µg per 100-500 µg of total proteins) and rotated at 4 °C, overnight. The immobilized agarose beads were washed at least three times with an immunoprecipitation (IP) lysis buffer (Thermo Scientific, Rockford, IL), and then boiled for 5 min. at 95 °C before a sodium dodecyl sulfate (SDS) buffer was added. Equal volumes of supernatants were used for western blotting analysis.

### Purification of protein

Full lengths of the transmembrane domain (1-40), and the prohibitin domain (41-173) of mouse PHB were tagged with 6x histidine tags. The coiled-coil domain (174-272) of mouse PHB was tagged with a 10x histidine tag (28). Competent *Escherichia coli* cells transformed with the appropriate pET28a plasmids were grown and induced using an isopropyl β-D-1-thiogalactopyranoside system. After centrifugation, cells were lysed, and supernatants were purified by fast protein liquid chromatography using a nickel-nitrilotriacetic acid affinity column. After purification, dialysis was performed with a storage buffer (10 mM Tris-HCl, 1 mM EDTA, PH 8.0, 50% glycerol).

### Pull-down assay

Equal amounts of whole cell extracts and purified proteins were rotated with a poly-histidine antibody at 4 °C, overnight. Then, protein A plus agarose beads were added and rotated for 1 h at 4 °C. The immobilized agarose beads were washed at least three times with an IP lysis buffer, and SDS buffer was added. The beads were boiled for 5 min. at 95 °C. Equal volumes of supernatants were used for western blotting analysis.

### Western blotting

Cells were lysed in a Pierce IP lysis buffer (Thermo Scientific, USA) containing a protease inhibitor cocktail (Sigma, Louis, MO) and 1 mM phenylmethanesulfonyl fluoride (PMSF, Sigma, St. Louis, MO) at 4 °C for 20 min. The lysate was centrifuged at 13,000 rpm for 20 min and the supernatant was obtained. The protein of the supernatant was quantified using a BCA Protein Assay kit with BSA as the standard. Equal amount of proteins was separated by NuPAGER 10% bis-tris gel (Invitrogen, CA, USA) and transferred onto a polyvinylidene fluoride transfer membrane.

β-actin antibody (1:10,000, GeneTex, GTX629630), Myosin-Va antibody (1:800, Cell Signaling Technology, Beverly, MA, 3402S), Rab27a antibody (1:500, Santa Cruz, CA, sc-74586), Mlph antibody (1:600, Protein Tech Group, Inc, Chicago, IL, 10338-1-AP), MADD antibody (1:700, Thermo Scientific, USA, PA5-19879), PHB1 antibody (1:500, Invitrogen, CA, MA5-12858), PHB2 antibody (1:500, Invitrogen, PA1-29883), poly-histidine antibody (1:5,000, Sigma, St. Louis, MO, USA, H1029), FLAG antibody (1:1,000, Sigma, St. Louis, MO, USA, F1804) and T7 antibody (1:10,000, Novagen, Germany, 69522-3) were kept at 4 °C overnight. Horseradish peroxidase conjugated for anti-rabbit (1:80,000, Sigma, St. Louis, MO, USA, A0545) or anti-mouse (1:20,000, Sigma, St. Louis, MO, USA, A9044) were used at 4 °C for 2 h. Binding antibodies were detected using an SuperSignal^TM^ West Pico PLUS Chemiluminescent substrate (Thermo Scientific, USA). The bands on the membrane were detected with chemiluminescence and visualized using FluorChem E (Biocompare, USA).

### Statistical Analysis

Differences between two groups were analyzed using the Student *t* test. To compare three groups, we performed ANOVA followed by Tukey-Kramer post hoc test. P<0.05, P<0.01, P<0.001 indicates a statistically significant difference.

## Supplementary Material

Supplementary figures.

## Abbreviations

PHB: Prohibitin
Mlph: Melanophilin
Myo Va: Myosin Va
PLA: Proximity Ligation Assay
co-IP: co-immunoprecipitation
