# Supplementary material for: A novel function of Prohibitin on melanosome transport in melanocytes
Source: Theranostics. 2020 Mar 4;10(9):3880–91. doi: 10.7150/thno.41383 (PMC7086355; doi:10.7150/thno.41383)
Supplement: Supplementary file 1 — Supplementary figures. [file thnov10p3880s1.pdf]

**Figure S1**

**A**

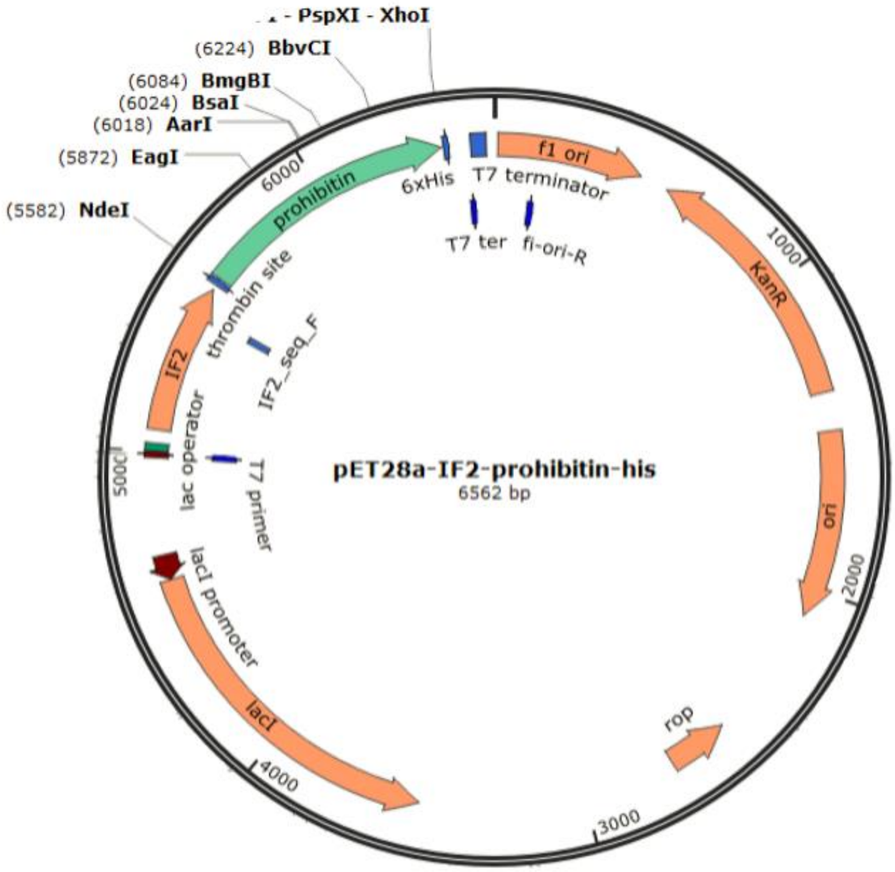

PspXI - XhoI
(6224) BbvCI
(6084) BmgBI
(6024) BsaI
(6018) AarI
(5872) EagI
(5582) NdeI
6000
prohibitin
6xHis
T7 terminator
f1 ori
T7 ter
fi-ori-R
1000
KanR
thrombin site
IF2_seq_F
IF2
lac operator
5000
T7 primer
pET28a-IF2-prohibitin-his
6562 bp
ori
2000
lacI promoter
lacI
rop
4000
3000

B

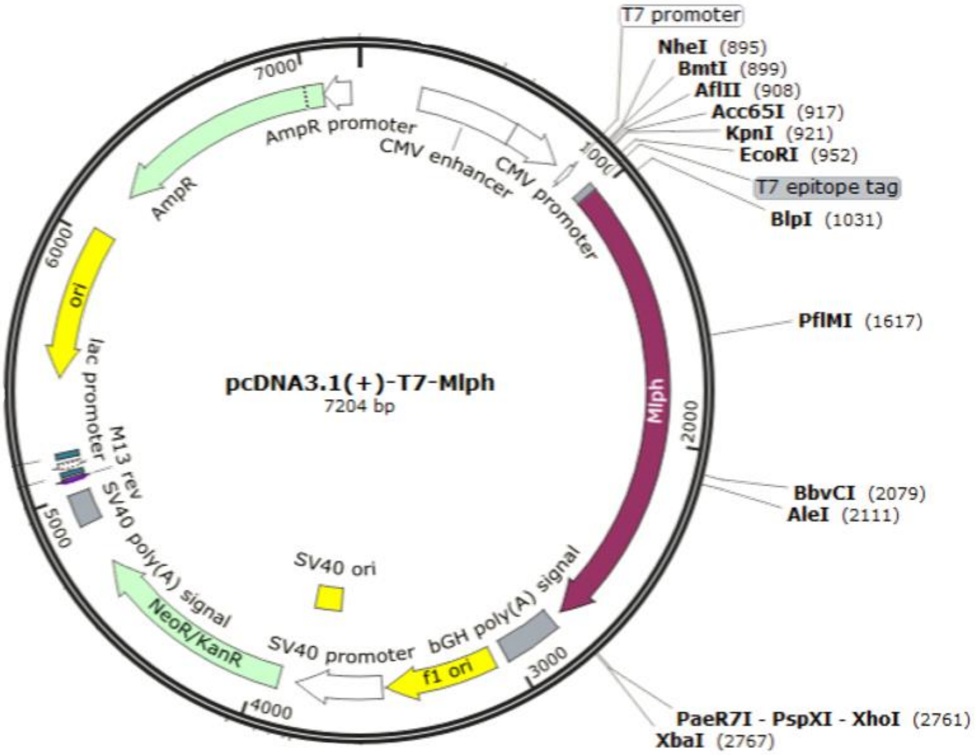

T7 promoter
NheI (895)
BmtI (899)
AflII (908)
Acc65I (917)
KpnI (921)
EcoRI (952)
T7 epitope tag
BlpI (1031)
PflMI (1617)
BbvCI (2079)
AleI (2111)
PaeR7I - PspXI - XhoI (2761)
XbaI (2767)
pcDNA3.1(+)-T7-Mlph
7204 bp
Mlph
CMV enhancer
CMV promoter
AmpR promoter
AmpR
ori
lac promoter
M13 rev
SV40 poly(A) signal
NeoR/KanR
SV40 ori
SV40 promoter
bGH poly(A) signal
f1 ori
1000
2000
3000
4000
5000
6000
7000

**C**

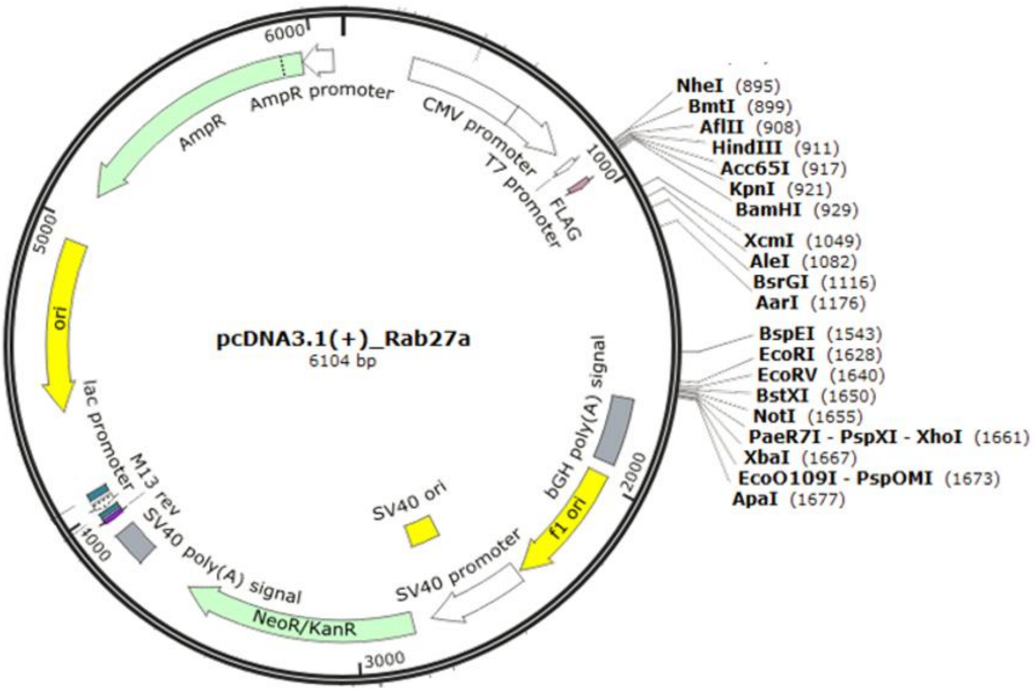


**A, B, C** Mouse PHB(**A**), Mlph(**B**), Rab27a(**C**) plasmids were designed and constructed

**Figure S2**

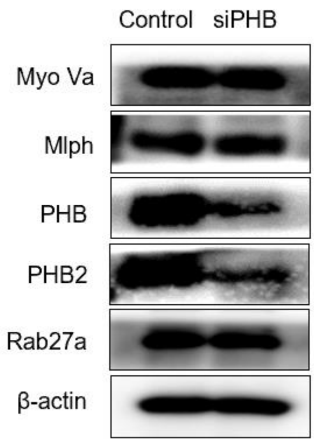


Validation of *si*PHB. IB analysis for Rab27a, PHB, Mlph, and Myosin-Va after transfection of *si*PHB (20 nM) for 72h.
